# Prevalence of Peripheral Arterial Disease among Adult Patients Attending Outpatient Clinic at a General Hospital in South Angola

**DOI:** 10.1155/2016/2520973

**Published:** 2016-05-15

**Authors:** Feliciano Chanana Paquissi, Arminda Bimbi Paquissi Cuvinje, Almeida Bailundo Cuvinje

**Affiliations:** ^1^Department of Medicine, Clínica Girassol, Luanda, Angola; ^2^General Hospital of Huambo, Huambo, Angola; ^3^José Eduardo dos Santos University, Huambo, Angola

## Abstract

*Background*. Peripheral arterial disease (PAD) is a common manifestation of atherosclerosis, whose prevalence is increasing worldwide, and is associated with all-cause mortality. However, no study has assessed this disease in Huambo. The aim of this study was to evaluate the prevalence of PAD in patients attending an outpatient clinic at a general hospital in Huambo, South Angola.* Methods*. A cross-sectional study, including 115 patients aged 40 years and older attending an outpatient service. The evaluation included a basic questionnaire for lifestyle and medical history and ankle-brachial index (ABI) measurement using hand-held Doppler. PAD was defined as an ABI ≤0.9 in either lower limb.* Results*. Of 115 patients, 62.60% were women with a median age of 52.5 (range of 40 to 91) years. The prevalence of PAD was 42.6% (95% confidence intervals [CI]: 95%: 33.91–52.17%). Among patients with PAD, 95.92% had mild disease and 4.08% moderate to severe disease. The main risk factor for PAD was age (≥60 years) (*χ* ^2^ = 3.917, *P* ≤ 0.05). The prevalence was slightly higher in men and hypertensive subjects, but without statistical significance with ORs of 1.5 (95% CI: 0.69–3.21) and 1.42 (95% CI: 0.64–3.17), respectively. Hypertension was also high in the group (66.95%).* Conclusion*. The prevalence of PAD was 42.6%, higher in those aged 60 years and older. More studies, with representative samples, are necessary to clarify PAD prevalence and associated risk factors.

## 1. Introduction

Peripheral arterial disease (PAD) is an important manifestation of atherosclerosis, which in 2010 was estimated to affect more than 202 million people worldwide [1]. From 2000 to 2010, the number of people with the disease increased by 28.7% and 13.1% in low- and high-income countries, respectively [1]. It affects about 4.3% of Americans aged 40 years and older [2], reaching 12.29% and 29% in those over 60 and 70 years of age, respectively [3, 4].

The presence of PAD is an important marker of cardiovascular risk [5, 6] and is associated with increased all-cause and cardiovascular mortality [7–9]. PAD is independent risk factor for several other negative outcomes such as left ventricular hypertrophy and heart failure [10, 11]; it increases twice the risk of left main coronary artery and multiple vessels involvement, in patients with coronary artery disease (CAD) undergoing coronary angiography [12], and, by functional limitations it imposes, is associated with depression and loss of quality of life [13, 14].

The main risk factors are advanced age (>65 years), smoking, diabetes, hypertension, and chronic kidney disease [3, 15, 16]. Other risk factors are dyslipidemia, vitamin D deficiency, hyperhomocysteinemia, female sex, and black race/ethnicity [16–19].

In sub-Saharan Africa, there are few studies about PAD; however, between 1990 and 2010, the PAD was, with atrial fibrillation, the factor with the highest relative increase in the burden of cardiovascular diseases in the region [20]. The prevalence of PAD in different studies has varied depending on the age of the group, the presence or absence of risk factors like diabetes and hypertension in the studied population, and the diagnostic test used. Numerous studies have shown the black ethnicity as a risk factor for PAD, increasing twice the risk and affecting younger patients, even when adjusted for other cardiovascular risk factors that also occur with higher prevalence in blacks than other races [2, 21]. In a study with patients with an average age of 62.4 years, at South Africa, the prevalence of PAD was 29.3% [22]; and in another study with diabetic patients, in Nigeria, the prevalence was higher reaching 52.5% [23], showing a prevalence significantly high compared to studies with other populations of the same average age.

Despite a high prevalence, and being CAD risk equivalent, these patients are often overlooked, and only a quarter of patients are submitted to treatment [4, 24, 25]. In Angola, particularly in Huambo, no study was found about the prevalence of PAD. The aim of this study was to assess the prevalence of PAD in patients attending an outpatient clinic at South Angolan hospital.

## 2. Materials and Methods

### 2.1. Type and Population of The Study

This is a cross-sectional study, with 115 patients aged 40 years and older, seen at the outpatient medical care service in the General Hospital of Huambo, in September 2015. Huambo is a province of southern Angola, with an area of 34.274 km^2^ and a population of ~1.8 million inhabitants [26]. The General Hospital of Huambo is a tertiary unit, whose outpatient medical care service attends on average 2.4 thousand patients a month, aged 18 years and older. Despite being conceived as a tertiary level hospital, most patients are of primary care profile and seek assistance by spontaneous demand due to subacute or mild symptoms, which under normal conditions would be assessed at basic health units. However, due to weak structuring of the basic health services, there is this flow diversion to the hospital of greater complexity. Since they do not meet criteria to be attended in the emergency department, they are evaluated at the outpatient service. Therefore, most patients seek assistance without a previously diagnosed chronic disease, for follow-up, and are seen in general medicine consultation. Among patients with previous diagnosis are those with surgical pathology in evaluation for elective surgery and those in postorthopedic trauma follow-up.

For the study, all adults aged 40 years and older that appeared for consultation during the period of data collection (in September) were invited to participate. Those with any difficulty of measuring blood pressure in 4 limbs were excluded. Data of 118 patients were collected, but 4 of them were excluded due to incomplete or erroneous data. So, the final analysis included a total of 115 patients. Despite a considerable average number of consultations per month, the sample was small because most people seeking care were below 40 years, reflecting the age pyramid of the population in the country, where children and young adults are the majority. Another reason is that, due to the significant burden of trauma in the population, a considerable number of subjects in postorthopedic trauma follow-up were excluded due to the difficulty of measuring blood pressure in 4 limbs, in the presence of orthotics and synthetic material.

### 2.2. Study Variables

Data collection included a basic questionnaire with open-ended questions, applied by trained interviewers, consisting of basic questions on the following variables: age, sex, smoking history, personal history of diabetes, hypertension, cerebrovascular or coronary artery disease, and use of medications.

The vascular examination was performed with the patient in the supine position, after at least 10 minutes of rest. Systolic blood pressure (SBP) was measured in brachial artery of each upper limb and at the dorsal or posterior tibial arteries in lower limbs, using hand-held vascular Doppler (BT 200V, 8 MHz; Bistos Co., Ltd., Korea) and a sphygmomanometer calibrated and certified by the National Institute of Metrology, Quality, and Technology (INMETRO) of Brazil. The ankle-brachial index (ABI) was calculated by dividing SBP on each lower limb by the higher SBP in the arm, according to the specific guidelines [27, 28]. PAD was defined by an ABI ≤0.9 or greater than 1.3 in at least one lower limb or prior revascularization for PAD [4, 19]. Subjects with PAD were stratified into three categories of severity according to the value of the ABI: mild (ABI between 0.70 and 0.9); moderate (ABI between 0.4 and 0.69); and severe or critical limb ischemia (ABI < 0.4).

### 2.3. Statistical Analysis

The prevalence of PAD was described in absolute and relative numbers, and continuous variables were expressed as median. The prevalence of PAD was calculated in the overall group, specific to age and sex, with respective 95% confidence intervals. Comparison between categorical variables was examined using chi-square, adopting a level of significance for *P* < 0.05. All statistical analyses were performed using a public domain statistical program (OpenEpi version 3.03a).

### 2.4. Ethics and Data Collection

The study procedures were performed only after informed verbal consent was obtained from the participants or those responsible for them, without any record, because considerable proportion was illiterate, so they do not know how to sign. The confidentiality of patient identification and individual data was guaranteed. All research procedures, including verbal consent procedure, were evaluated and approved by the Scientific and Pedagogical Board of the hospital, that is, the organ responsible for ethical issues in research at the institution, in the absence of formal ethical committee in the province.

## 3. Results

A total of 115 patients were included, the median age was 52.5 years, 62.60% were women, and all subjects were Africans and blacks. Baseline demographic and clinical characteristics of patients are presented in Table 1.

**Figure 1:**
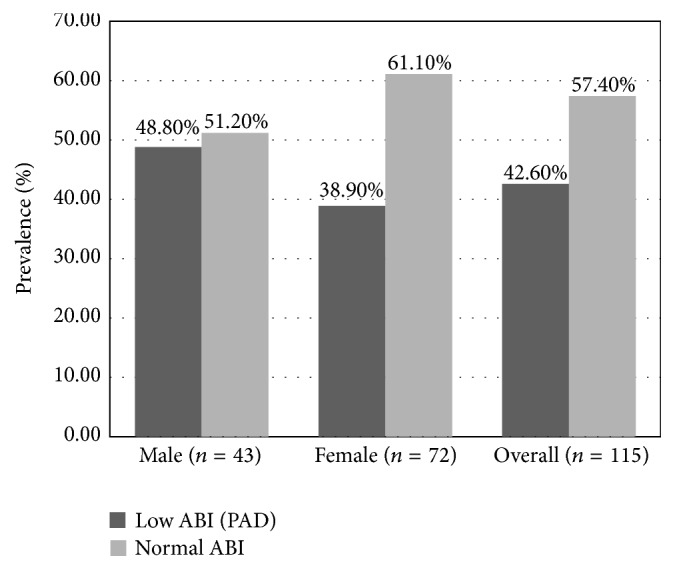
Overall, and by gender prevalence of PAD in adults aged 40 years and older. General Hospital of Huambo, Angola, 2015 (*n* = 115).

The prevalence of peripheral arterial disease (ABI ≤ 0.9) was 42.6% (*n* = 49; 95% CI: 33.91–52.17%) (Figure 1); and nobody had an ABI value >1.3. Among patients with PAD, 95.92% had mild disease and 4.08% had moderate to severe disease. In multivariate analysis, the occurrence of PAD was significantly associated (*χ* ^2^ = 3.917, *P* ≤ 0.05) with age of 60 years and older (60% versus 37.78%). There was no significant association between PAD and hypertension (OR: 1.42; 95% CI: 0.64–3.17) or male sex (OR: 1.5; 95% CI: 0.69–3.21) (Table 2 and Figure 1).

In the group, 66.95% (*n* = 77; 95% CI: 58.26–76.52%) had hypertension; 13.04% (*n* = 15; 95% CI: 6.95–20%) had prehypertension; 6.95% (*n* = 8; 95% CI: 2.60–12.17%) self-reported diabetes, and 9.56% (*n* = 11; 95% CI: 3.34–15.65%) reported active smoking. Nobody reported personal history of cerebrovascular or coronary artery disease. Among hypertensive patients, only 35.06% were in treatment and 10.38% were with controlled blood pressure (<140/90 mm Hg), with the most commonly used medications being the renin angiotensin aldosterone system inhibitors, either alone or in combination with diuretics and/or calcium channel antagonists. None of the participants were taking statin or clopidogrel.

## 4. Discussion

In this study, we found prevalence of PAD to be 42.6%, which is more in accordance with Africans studies made in populations with specific risk factors such as diabetes in Uganda (39%) [29] and Nigeria (40%) [30] and hypertension in Nigeria (41.8%) [31]. However, it is relatively greater than that found in general population studies as that done in Brazzaville (32.4%) [32], especially considering that the population of this study is of general demand, which should actually be seen in primary care; and considering the average age in this study, prevalence is even higher compared to American populations of same age [2]. There are no other studies with our population to compare with directly, since this is the first study on PAD in the country.

This high prevalence may reflect several factors, such as the racial role in early arterial stiffness [21], to PAD as a target organ damage reflecting misdiagnosis, low treatment, and control of its main risk factors such as hypertension and diabetes [33, 34]. This is evident looking at the fact that the prevalence of hypertension in the group was high (66.95%), and of these only 35.06% and 10.38% were in treatment and had their blood pressure controlled, respectively. This shows how late health professionals are arriving at diagnosing, treating, and controlling risk factors, when PAD and other target organs' damage are already installed, which is in concordance with that high prevalence found among hypertensive patients in Nigeria [31]. The prevalence of self-reported diabetes was 6.95% but may be underestimated too, as shown in the study where almost 42.8% of diabetics were unaware of their status [35].

Unfortunately, it was not possible to study other risk factors for atherosclerosis such as lipid levels, hyperhomocysteinemia, vitamin D deficiency, and some inflammatory markers that could help us understand the reason for such a high prevalence of PAD as found in this study.

In this study, we highlight importance of knowing about the disease as a CAD risk equivalent and independent marker for several clinical outcomes such as increased total and cardiovascular mortality, number of coronary events, and stroke [5, 6, 8, 36], risk of amputation with all social and psychological consequences [14], and even risk of developing dementia, as demonstrated in a study population of Central Africa [37]. So, with a simple test (ABI), we could have a window to access the individual cardiovascular and no vascular health [36]. This importance is enhanced because such knowledge would justify changes in patient's approach such as the introduction of statins and platelet antiaggregant for those who were not taking any of these drugs [19, 38] and even more rigorous blood pressure target (<130/80 mm Hg) in individuals who are diagnosed with this CAD risk equivalent [39]. In the present study, no patient with PAD was taking statins or clopidogrel, which shows how the lack of diagnosis was a substrate for the patients remaining without this treatment whose benefit is well established in disease [19, 38].

In addition, it is important to emphasize the role of race as a risk factor in the disease, since the black race is associated with increased progression from normal to low ABI [40] and higher prevalence [2], which may explain, in part, a high prevalence found in a relatively young population. Also black patients develop greater need for reintervention and amputation after endovascular procedures [41], longer hospital stay after bypass [42], and higher cardiovascular mortality [43], even after adjusting for other factors, which reflects not only differences in access to health, but a possible role of race as an independent pathophysiological and prognostic factor [42].

## 5. Conclusions

This is the first study on PAD in Huambo and in the country. So, a high prevalence found in this study, despite being with a no representative sample, should be at least a starting point for more studies on the subject to clarify PAD prevalence and associated risk factors.

## Figures and Tables

**Table 1:** Demographical and clinical characteristics of the study population.

Characteristics	*n* = 115
Age in years, median (range)	52.5 (40–91)
Female gender (%)	62.60
Peripheral arterial disease (%)	42.6
Systemic arterial hypertension (%)	66.95
Self-reported diabetes (%)	6.95
Smoking (%)	9.56

Medical therapy (%)	
Angiotensin-converting enzyme inhibitor or angiotensin receptor blockers	17
Calcium channel antagonists/diuretics	12/4
Acetylsalicylic acid	4
Statins/clopidogrel	0/0

**Table 2:** Univariate analysis of risk factors associated with peripheral arterial disease in adults of 40 years of age and older, General Hospital of Huambo, Angola, 2015 (*n* = 115).

Factor	OR	95% CI	*P* value
Age (≥60 versus <60 years)	3.917^*∗*^	—	0.04
Sex (male versus female)	1.5	0.69–3.21	0.39
Hypertension	1.42	0.64–3.17	0.49

OR: odds ratio; CI: confidence interval. ^*∗*^Chi-square.

## Abbreviations

ABI: Ankle-brachial index
CAD: Coronary artery disease
PAD: Peripheral arterial disease
SBP: Systolic blood pressure.

## Additional Points

This is a single center and hospital-based study; so it does not represent the general population of Huambo, even with study population do not present the profile of a tertiary hospital patients. There was no possibility to study the associated risk factors.
